# Can We Modulate Our Second Brain and Its Metabolites to Change Our Mood? A Systematic Review on Efficacy, Mechanisms, and Future Directions of “Psychobiotics”

**DOI:** 10.3390/ijms26051972

**Published:** 2025-02-25

**Authors:** Stefania Garzone, Ioannis Alexandros Charitos, Manuela Mandorino, Maria Elena Maggiore, Loredana Capozzi, Bujar Cakani, Gabriel César Dias Lopes, Luisella Bocchio-Chiavetto, Marica Colella

**Affiliations:** 1 Interdisciplinary Department of Medicine, Section of Microbiology and Virology, School of Medicine, University of Bari, 70124 Bari, Italy; s.garzone@studenti.uniba.it (S.G.); m.mandorino3@studenti.uniba.it (M.M.); m.maggiore6@studenti.uniba.it (M.E.M.); loredana.capozzi@izspb.it (L.C.); 2 Istituti Clinici Scientifici Maugeri IRCCS, Pneumology and Respiratory Rehabilitation Unit, “Institute” of Bari, 70124 Bari, Italy; 3 Istituto Zooprofilattico Sperimentale della Puglia e della Basilicata, 71121 Foggia, Italy; 4 Department of Clinical Disciplines, “Alexander Xhuvani” University of Elbasan, 3001 Elbasan, Albania; bujar.cakani@uniel.edu.al; 5 Department of Neuroscience and Mental Health, School of Science of Health, Logos University International (UNILOGOS), Miami, FL 33137, USA; president@unilogos.edu.eu; 6 Department of Neuroscience and Mental Health, School of Science of Health, European International University, 75018 Paris, France; 7 IRCCS Centro San Giovanni di Dio Fatebenefratelli, 25125 Brescia, Italy; luisella.bocchiochiavetto@uniecampus.it; 8 Department of Theoretical and Applied Sciences (DiSTA), eCampus University, 22060 Novedrate, Italy

**Keywords:** probiotics, gut-brain axis, neurotransmitters, psychiatric disorders, neurodevelopmental disorders

## Abstract

Psychobiotics, live microorganisms that provide mental health by interacting with the gut microbiota, are emerging as a promising therapeutic option for psychiatric and neurodevelopmental disorders. Their effectiveness in addressing conditions such as depression, anxiety, insomnia, stress, autism spectrum disorder (ASD), and eating disorders were examined through a comprehensive analysis of existing studies up to the first half of 2024, based on data from reliable electronic databases. We found that psychobiotics can significantly reduce symptoms of various psychiatric disorders by influencing neurotransmitter levels, regulating the hypothalamic-pituitary-adrenal (HPA) axis, and improving gut barrier function through short-chain fatty acids (SCFAs) and other metabolites. However, several limitations were identified, including inconsistent study methodologies, small sample sizes, and a lack of data on long-term safety. Addressing these limitations through rigorous research is essential for establishing standardized protocols and fully confirming the therapeutic potential of psychobiotics. In conclusion, psychobiotics show great promise as complementary treatments for mental health conditions, but continued research is necessary to refine their application and integrate them into clinical practice effectively.

## 1. Introduction

The term “probiotic” originates from the ancient Greek “pro-bios” which means “in favour of life”; therefore, its nature and benefits are partially revealed by its etymology. The first formal description of a probiotic dates back to 1908 and was attributed to Metchnikoff, who observed that individuals living in a certain region of Bulgaria had a longer lifespan than others living in other regions. This anecdote was correlated to the regular consumption by longer-lived individuals of a fermented milk product. Currently, the term probiotic refers to a living organism that, when ingested in adequate amounts, is able to cause beneficial effects in the host [1]. In 2013, Dinan et al. used the term “psychobiotics” to designate a subset of probiotics with potential applications in the treatment of psychiatric diseases [2]. Psychobiotics, indeed, can be beneficial microorganisms (probiotics) able to influence “gut-brain axis”. This term refers to a morpho-functional axis characterized by a bidirectional connection between the intestine, more precisely the enteric nervous system, and the central nervous system. Besides contributing to the proper function of the digestive tract and to nutrition, the gut microbiota is responsible for regulating the immune system and the central nervous system, thereby facilitating a state of well-being [3]. Several neuroactive molecules, such as gamma-amino butyric acid (GABA), acetylcholine, serotonin, and catecholamines produced physiologically by every person are also derived from microbial by-products, and many of them have been isolated from bacteria in the human gut. Neurotransmitters, which are secreted by bacteria into the intestinal lumen, can stimulate the epithelial cells to release some molecules which, in turn, can regulate neural signaling into the enteric nervous system and thus can regulate the brain function and the host behavior [4]. Maintaining a diverse and balanced composition of gut microbiota is critical for the homeostasis of the gut-brain axis. Disruption of this balance, known as dysbiosis, caused by stress, antibiotics, or environmental factors, can alter neurotransmitter levels and disrupt stress responses [5]. Stress-induced changes in gut microbiota composition, often associated with increased gut permeability (“leaky gut syndrome”), amplify neuroinflammatory responses [6]. This allows bacterial products like lipopolysaccharides (LPSs) to enter systemic circulation, triggering immune responses and the release of pro-inflammatory cytokines. Studies using germ-free (GF) animal models have shown that the absence of gut microbiota leads to increased stress responses and abnormal behaviors, highlighting the microbiota’s role in regulating neurodevelopment and behavior [7].

The research on psychobiotics is still at the beginning; indeed, none of the regulatory body, such as Food and Drug Administration (FDA) or European Food Safety Authority (EFSA), has defined specific claims for these particular probiotics. Nevertheless, this field is promising and rapidly growing; indeed, a large amount of studies are conducted for defining the mechanisms of action and the benefit of their supplementation in psychiatric pathologies, psychological disorders, and psychopathological conditions. Such interventions, which offer an interdisciplinary hint, will bring in the future a better awareness for the use of psychobiotics as a therapeutic support.

The discovery of the bidirectional link between the brain and the gut dates back to the early 19th and 20th centuries. Cannon’s study in the early 20th century showed that certain parts of the gastrointestinal system were sensitive to some mental status factors such as interest and attention, while Baumont, monitoring a patient’s stomach, observed an association between gastric secretions and some mood traits [8].

The communication between the gut microbiota and the central nervous system occurs first through the vagus nerve involving neuro-immune-endocrine mediators; this innervation contributes to sensory and motor functions (e.g., satiety, nausea, pain, etc.). Another important communication channel is the production of short-chain fatty acids (SCFAs) such as butyrate, propionate, and acetate, which represent the metabolic product of the gut microbiota components.

SCFAs have pleiotropic effects, and among their functions, we can mention: The regulation of the gut microbiota composition, while the intestinal barrier system regulates immunomodulation and anti-inflammatory activity; the activation of the production of prostaglandins E1 and E2, which improve the secretion of mucins thus providing mucosal protection against infections; the production of antimicrobial compounds such as peptides and bacteriocins, which fight the intestinal dysbiosis; the promotion of the production of IL-10 and IL-18; epigenetic regulation; the modulation of neural inflammation; the control of brain metabolism; the maintenance of the blood-brain barrier; the development of the neural plate [4].

Furthermore, it has been demonstrated that intestinal dysbiosis contributes directly to the alteration of several pathways related to synaptic plasticity, neuronal growth and repair, memory and learning processes [9]. By regulating inflammation and oxidative stress, SCFAs promote a good mitochondrial function and maturation of microglia, which prevents cognitive decline and the onset of neurodegenerative diseases. Some studies argue that persistent stress represents a significant risk for the onset of neuropsychiatric diseases and, in particular, Cruz-Pereira et al. found that the gut-brain axis is crucial in the link between stress and the brain [10].

Several clinical studies demonstrate that SCFAs regulate various processes at the basis of central nervous system (CNS) diseases such as oxidative stress, inflammatory response, neuronal apoptosis, and the integrity of the blood-brain barrier (BBB).

In fact, both the peripheral and central nervous systems are made up of SCFA receptors that can be found at the neuronal level. It has been shown that SCFAs can stimulate the central part of the colon to produce serotonin (5-HT); instead, the acetic acid can alter the action of serotonin by decreasing the expression of the serotonin receptor 5-HT3B. In addition, increased levels of SCFA have been linked to dietary fibers and amino acids needed for the production of serotonin in the gastrointestinal tract, a process that contributes to the preservation of mental health.

Finally, SCFAs bind to intestinal glucagon-like peptide 1 (GLP-1), and peptide YY sends signals to the brain via the vagus nerve and bloodstream. This interaction affects aspects such as memory, learning, and mood [11].

The most frequently used probiotics are bacteria from the *Bifidobacteriaceae* and the families that once belonged to the *Lactobacillaceae*. Among the bacteria composing psychobiotics, the most frequent are the following: *Lactobacillus acidophilus*, *Bifidobacterium bifidum*, *Lactobacillus helveticus* R0052, *Lacticaseibacillus rhamnosus* R0011, *Bifidobacterium longum* R0175, *Lacticaseibacillus casei* strain Shirota.

The therapeutic potential of psychobiotics ranges from mild conditions such as anxiety and stress to severe disorders such as depression and autism [8]. The psychophysiological effects produced by these bacteria mainly comprise the following: psychological effects on emotional and cognitive processes; systemic effects on the HPA axis and on the response to stress and inflammation; neural effects on neurotransmitters and proteins [12].

Most psychobiotic research is carried out using animal studies, usually rodents, and are based on stress inductions and behavioral tests. For example, experiments performed on rodents showed that behaviors related to anxiety and depression are normalized after chronic psychobiotics supplementation. In particular, the *Bifidobacterium longum* 1714 strain improves behavior, cognition and physiological response in stressed mice, while *Lacticaseibacillus rhamnosus* JB1 lowers corticosterone levels caused by anxiety in the Elevated Plus Maze (EPM), and by despair in the forced swim test [8].

Psychobiotics can also help in the treatment of insomnia; in fact, several studies have shown an improvement in NREM (Non-Rapid Eye Movement) sleep during the rest phase and a notable improvement in the effectiveness of sleep and awakening episodes.

Patients suffering from ASD have been associated with altered levels of *Bacteroides*, *Firmicutes*, *Prevotella* and *Clostridium*, which can be corrected through the administration of psychobiotics. Furthermore, the analysis of stool samples from patients with ASD has shown an alteration of SCFA and, although the role of these acids is still not clear when associated to autistic symptoms, some studies have shown that using prebiotics, such as butyrate, improves symptoms in a mouse model of ASD [8].

Several studies demonstrate the beneficial effects of psychobiotics concerning eating behaviors, nutrition, and eating disorders. Certain overweight individuals, who underwent weight loss, showed a decrease in binge eating tendencies, disinhibition and food cravings, after *L. rhamnosus* HA-114 supplementation [13]. Similarly, probiotic supplementation with *Lactobacillus acidophilus* NCFM and *Bifidobacterium lactis* Bi-07—started seven days after bariatric surgery—has shown a reduction in the symptoms of food addiction and uncontrolled eating such as binge eating, associated to a sense of anguish and regret [14].

The present study aims to compare different clinical trials and randomized controlled-trials in order to evaluate the effectiveness of the use of psychobiotics in the following pathologies: depression, anxiety, mild cognitive impairment, mood and eating disorders, insomnia, stress, and autism spectrum disorder.

## 2. Methods

The research has been conducted in the first half of 2024 in reputed electronic databases (PubMed, the Cochrane Library, Scopus and Web of Sciences), retrieving 21 articles about the study subject. In all databases, we used the keywords “psychobiotic”, “psychobiotic AND synbiotics”, “probiotics AND mood disorders”, “probiotics AND psychiatry”, “probiotics AND autism”, “probiotics AND nutrition disorders”. The systematic review has been prepared according to the PRISMA rules and is registered in OSF Registries (Registration: https://doi.org/10.17605/OSF.IO/ATHNW).

**Figure 1:**
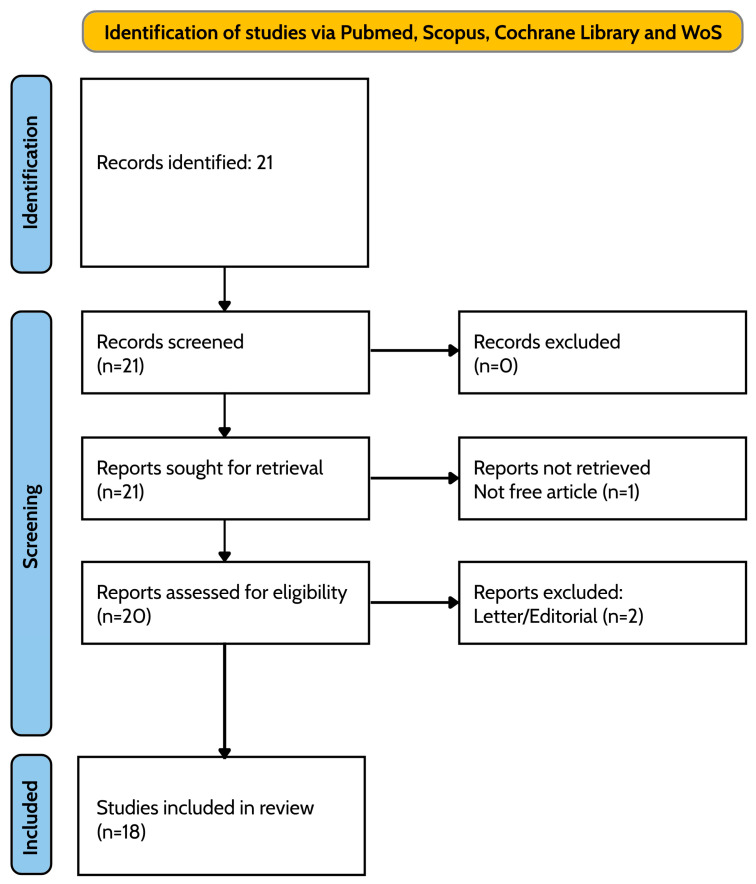
PRISMA flow diagram of study screening and selection.

Two groups independently screened the titles and abstracts and full-texts, excluding papers written in languages other than English, not accessible full-texts, and non-research articles. Only articles with human clinical trials were included and considered. A third author, expert in neuropsychiatry or psychology, supervised them to avoid possible misunderstandings, and at the end only 18 papers fitting with the aim and the criteria of inclusion and exclusion were considered (as shown in Figure 1).

Potential biases were defined with the JBI tool (https://jbi.global/critical-appraisal-tools, accessed on 6 December 2024). The results from all the included articles were systematically summarized and reported in the following section.

## 3. Results and Discussion

The search performed on the electronic databases, applying the filter on clinical trial and randomized controlled trial, generated only 21 articles since it is a recent and emerging topic and the first clinical trial was published in 2016. However, we excluded letters, articles without full-text, and a paper, which used the diet as an instrument for intestinal eubiosis; we selected only 18 articles, as shown in Figure 1.

The most studied microorganisms that constitute psychobiotics are certainly *Lactiplantibacillus plantarum*, *Lactobacillus acidophilus*, *Lactobacillus helveticus*, *Bifidobacterium longum*, *Limosilactobacillus reuteri*, and *Lactococcus lactis*. They are usually formulated in tablets, with a single or more microorganisms, but sometimes they are tested also in probiotic oral suspension (POS), which are made up of more bacterial strains. In every selected clinical trial, researchers resort to the use of questionnaires, with the aim to test the efficacy of the intervention. The most used questionnaires are Symptoms checklist (SCL-90), Perceived Stress Scale (PSS-10), State and Trait Anxiety Index (STAI), Hamilton Depression Rating Scale (HAM-D 17), Hamilton Anxiety Assessment Scale (HAM-A 14), and Short Form Health Survey (SF-36).

In this section, each manuscript will be analyzed in a subparagraph according to the target pathology for psychobiotic integration.

### 3.1. Depression

There is a large amount of evidence from preclinical and clinical studies that suggest that alterations in the gut microbiota, SCFA microbe-derived, D-amino acids, and other metabolites could play an important role in the pathophysiology of depression via the brain-gut-microbiota axis [15]. Major Depressive Disorder (MDD) represents the classical condition in this group of disorders and is characterized by five (or more) simultaneously present symptoms in a period of two weeks, such as depressed mood, mood and pleasure for daily life activities, loss or gain of weight, insomnia or hypersomnia, agitation, fatigue, reduced ability to think, concentrate or decide, recurring thoughts of death or suicidal ideation or suicide attempt. Such symptoms create clinically significant discomfort or impairment of the functioning of the social, working or other spheres, and constitute a diagnostic criteria. The selective serotonin reuptake inhibitors (SSRIs), serotonin–norepinephrine reuptake inhibitors (SNRIs), agomelatine, bupropion, vortioxetine and mirtazapine are the first-line recommended pharmacotherapy for MDD. Clinical features and medication characteristics influence the choice of an antidepressant. However, cognitive-behavioral psychotherapy represents the most efficient solution to face and recover from MDD [16].

Rudzki et al. [17] evaluated psychological and immunomodulatory consequences of *Lactiplantibacillus plantarum* 299v (LP 299v, 10 × 10^9^ CFU) supplementation in patients with MDD on therapy with SSRI, analyzing biochemical parameters, affective and cognitive functions. They randomized and assigned 79 patients to an 8-week double-blind, placebo-controlled trial. Members of the study were given either a SSRI with LP 299v or a SSRI with the placebo for 8 weeks. Sixty of those patients concluded the study and were examined: 30 participants were allocated to the LP 299v group, and 30 participants were allocated to the placebo group. HAM-D17, SCL-90, and PSS-10 were used to estimate the severity of psychiatric symptoms, meanwhile to investigate cognitive functions, the following were adopted: Attention and Perceptivity test (APT), Stroop test, Riff Figural Fluency test (RFFT), Trail Making test (TMT), and California Verbal Learning Test (CVLT). One of the aims was also to detect some biochemical parameters such as tryptophan (TRP), Kynurenine (KYN), Kynurenic acid (KYNA), 3-hydroxykynurenine (3HKYN), anthranilic acid (AA), 3-hydroxy anthranilic acid (3HAA), tumor necrosis factor-alpha (TNF-α), interleukin 6 (IL-6), interleukin 1-Beta (IL-1β), and cortisol plasma concentrations. As a primary outcome, measures executed with HAM-D 17, SCL-90, and PSS-10 reported no important changes in depressive and anxiety symptoms, and concerning the biochemical parameters, they did not find any considerable effect (except for KYN). In conclusion, the implementation of LP 299v to SSRI therapy enhanced cognitive performance and reduced KYN concentrations in MDD patients.

Karakula-Juchnowicz [18] analyzed the impact of a psychobiotic supplementation (consisting of *Lactobacillus helveticus* R0052 and *Bifidobacterium longum* R0175, 3 × 10^9^ CFU per day divided into two equal doses), a gluten-free diet and their combination in patients with MDD, examining their mental state, inflammatory and gut permeability markers, and gut barrier function. The 12-week, randomized, double-blind and placebo-controlled trial involved 120 adults with MDD divided into four groups:

(1) Probiotic supplementation+ gluten-free diet group (PRO-GFD; n. 30)
(2) Placebo supplementation+ gluten-free diet group (PLA-GFD; n. 30)
(3) Probiotic supplementation+ gluten-containing diet (PRO-GD; n. 30)
(4) Placebo supplementation + gluten-containing diet (PLA-GD; n. 30)

The primary outcomes were the intensity of depression symptoms using the Montgomery-Asberg Depression Rating Scale (MADRS) and Beck’s Depression Inventory (BDI), the severity of psychopathological deterioration using SCL-90, the living conditions using the SF-36, and stress measure using PSS-10. They estimated as secondary outcomes the levels of different biomarkers which include proinflammatory biomarkers (hs-Crp, IL-6, IL-1β, and TNF-α), gluten sensitivity biomarkers (anti-TG2 IgG and anti-AGA IgG/IgA), intestinal permeability biomarkers (I-FABP/FABP-2 and LBP), total cholesterol, LDL and HDL cholesterol, triglycerides, glucose and insulin, cortisol, ALT and AST. As a tertiary outcome, gut microbiota and SCFAs levels were analyzed in stool samples, brain activity was checked with electroencephalography (EEG), and gastrointestinal symptoms by the gastrointestinal symptom rating scale (GSRS). The study suggests that the combination of gluten-free diet and probiotic supplementation can stop the immune-inflammatory cascade in the MDD course and enhance both psychiatric and gut barrier-associated features.

Tian et al. [19] evaluated the psychotropic potential of *Bifidobacterium breve* CCFM1025 (a promising psychobiotic candidate strain, with a 10^10^ CFU concentration) in the treatment of MDD in a double-blind, randomized clinical trial. The study included 45 patients with MDD who were treated daily for four weeks: 20 patients received freeze-dried CCFM1025 and 25 patients, the placebo group, received maltodextrin. The psychiatric and gastrointestinal symptoms of the patients were assessed using questionnaires such as the Hamilton Depression Rating Scale-24 items (HDRS-24), MADRS, the Brief Psychiatric Rating Scale (BPRS) and GSRS; biological markers were also measured, including the determination of serum 5-HT and 5-hydroxyindoleacetic acid, cortisol, TNF-α and IL-β, by analyzing the faecal microbiome and metabolites of tryptophan metabolism. The endpoint HDRS-24 and MADRS scores were significantly reduced from baseline, highlighting a better antidepressant-like effect for the CCFM1025 group compared to the placebo group. Most likely through the regulation of the serotonergic system, emotional disturbances and gastrointestinal dysfunction based on BPRS and GSRS scores were also reduced in the CCFM1025-treated group. CCFM1025 was able to reduce serum serotonin turnover significantly and more extensively than the placebo, possibly due to changes in the gut microbiome and gut tryptophan metabolism under probiotic treatment.

### 3.2. Anxiety

Anxiety disorders include excessive fear, anxiety, and related behavioral disorders. Fear is the emotional response to an eminently real or perceived threat, while anxiety is the anticipation of a future threat. Panic attacks play an important role within anxiety disorders as a particular type of fear response. Anxiety disorders differ from normal evolutionary fears because they are excessive or persistent compared to the stage of development. Anxiety disorders differ from each other in the types of objects or situations that cause fear, anxiety or avoidance behavior, and the associated cognitive ideation. The main features of generalized anxiety are persistent and excessive in various areas, including work and school performances. In addition, the individual presents physical symptoms including restlessness or agitation, easy fatigue, difficulty in concentration or memory gaps, irritability, muscle tension and sleep disturbances. In the therapy of anxiety disorders, the most indicated and used psychotropic drugs are benzodiazepines, which are more effective in the short-term treatment and the possible cause of long-term dependence and tolerance. Cognitive psychotherapy and structured therapy consisting of psycho-education and relaxation training were effective, as well [16]. Moreover, a dysbiotic gut microbiota has been associated with the development of mental health and brain dysfunctions. In particular, adolescence is a sensitive period and changes during this phase, for example, dietary interventions, could lead to long-lasting consequences [20].

The study conducted by Colica et al. [21] investigates the effects of a particular psychobiotic formulation on body composition and anxiety. The POS under study contained strains of *Streptococcus thermophilus* I-1630, *Lactobacillus bulgaricus* I-1632 and I-1519, *Lactococcus lactis* subsp. *lactis* I-1631, *Lactobacillus acidophilus*, *Streptococcus thermophiles*, *Lactiplantibacillus plantarum*, *Bifidobacterium lactis* I-2494, and *Limosilactobacillus reuteri* DSM 17938. The suspension consisted of 1.5 × 10^10^ CFU for each bacterial strain. The study was conducted on 30 healthy patients between 21 and 72 years of age, comprising 83.3% women and 16.7% men, randomly divided into three groups: a group that received the oral suspension of psychobiotics (POSG); a diet treatment group (DTG) that followed a hypocaloric diet; and a combination treatment group (CTG) that was both treated with POS and a hypocaloric diet. The different treatments were carried out for 3 weeks and all patients underwent an anthropometric analysis (measurement of weight, height, body mass index “BMI”, waist and hip circumference and waist-to-hip ratio), bioelectrical impedance analysis (BIA), dual X-ray absorptiometry (DXA) and the HAM-A before and after the treatment. Significant decreases in the HAM-A, especially for the anxious subjects, were found for the entire study population after POSG and CTG treatments, and no effect was found on the HAM-A score of the patients in the DTG, who had only followed the diet without taking POS. Significant differences in weight, waist and hip circumference and body composition were found in the DTG, but not POSG. In the CTG, a greater change in TBFat loss and a significant reduction in android and gynoid fat mass were evidenced. These data underline the role of probiotics as a dietary supplement. In this study, taking POS for three weeks was a good approach considering the low number of patients involved.

In 2019, Tran et al. [22] performed a double-blind, placebo-controlled, randomized controlled scientific study on a group of 86 healthy college students (75.6 percent female) from Houston Baptist University, aged 18 to 31. Eligible participants were divided into a placebo group (group C) and four groups that were given over-the-counter probiotics of different composition: group A with 50 billion CFU and 18 bacterial species, group B with 50 billion CFU and 10 species, group D with 15 billion CFU and 18 species, and finally group E with 10 billion CFU and 10 bacterial species. The participants completed questionnaires, such as Beck anxiety inventory (BAI), anxiety control questionnaire-revised (ACQ-R), positive and negative affect schedule (PANAS), negative mood regulation (NMR), Penn state worry questionnaire (PSWQ), for evaluation before and after 28 days of daily intake. This study revealed that probiotics with high CFU values and the number of bacterial species were more effective in improving panic anxiety, neurophysiological anxiety, negative effect, worry and increased negative mood regulation. African/African American participants showed an even more significant decrease in negative effect. The results indicate that gender and ethnicity may represent a covariance with anxiety and warrant further investigation. It was also found in the study that participants with high distress reported more improvements than those with normal distress. The special feature of this study is the analysis of the effect of CFU and species count on the efficacy of probiotics.

The aim of Gualtieri et al. [23] clinical interventional study was to examine the combined effect of the single nucleotide polymorphism (SNP) of IL-1β rs16944 and probiotic administration on the phenotypes of mood disorders in a group of Italian patients. Previous studies had amply demonstrated that the expression of IL-1β, a circulating proinflammatory cytokine closely related to mood disorder symptoms, is strongly increased in the presence of the rs16944 polymorphism located in the promoter region. Ninety-seven patients (61.9% women), aged 18 to 62 years, were selected and randomly divided into two groups: probiotic oral suspension group (POSG) and placebo control group (PCG) and received the respective treatment for 12 weeks. The POS consisted of strains of *Streptococcus thermophilus*, *Bifidobacterium animalis* subsp. *lactis*, *Bifidobacterium bifidum*, *Lactobacillus bulgaricus*, *Lactococcus lactis* subsp. *lactis*, *Lactobacillus acidophilus*, *Lactiplantibacillus plantarum*, *Limosilactobacillus reuteri*. Each bacterial strain had a concentration of 1.5 × 10^10^ CFU. At time 0 (T0) and at the end of the treatment (T1), the psychological profile was assessed for all volunteers using HAM-A, Body Uneasiness Test (BUT), SCL-90R, and finally saliva samples were taken. Genotyping was performed on DNA extracted from salivary samples. After 12 weeks of intervention, a significant reduction in the total HAM-A score was detected in the POSG, compared to the PCG. Furthermore, IL-1 β carriers had a moderate risk of developing anxiety and in IL-1 β POSG carriers we observed a reduction in the HAM-A score. Probiotic consumption can modify anxiety symptoms, especially in healthy adults with the minor A allele of rs16944 as a risk factor. The particular results of this study certainly demonstrate the efficacy of probiotic use in mood disorders and suggest genetic association studies for the use of personalized therapies with psychobiotics.

The recent work conducted by Wu et al. [24] focused on evaluating the effects of psychobiotics on a group of highly stressed clinical nurses selected from a medical center in Northern Taiwan. For the treatment, 10 billion cells of a heat-killed (HK) strain of *Lacticaseibacillus paracasei* PS23 (HK-PS23) were used. The double-blind, randomized, placebo-controlled study included 65 nurses with a score of 27 or higher on the PSS-10 at the end of the selection process, i.e., with high levels of stress. Participants were randomized into two groups taking HK-PS23 or a placebo for 8 weeks. The initial and end-of-treatment values of PSS-10, Job Stress Scale, STAI, emotional questionnaires, gastrointestinal severity questionnaires, TMT, biological blood markers, and sleep data were analyzed. In particular, this study found that blood cortisol measurements decreased significantly in the HK-PS23 group after 8 weeks. Baseline and endpoint results at the end of the treatment of PSS, Patient Health questionnaire (PHQ-9), Job Stress Scale (JSS), STAI, insomnia severity index (ISI), emotional questionnaires, Quality of life enjoyment and satisfaction questionnaire short form (QLESQ-SF), visual analogue scale for gastrointestinal symptoms (VAS), TMT, biological blood markers, and sleep data were analyzed. In particular, this study found that blood cortisol measurements decreased significantly in the HK-PS23 group after 8 weeks. Further analysis on the subgroups that showed higher initial stress or anxiety (nurses with STAI ≥ 103) revealed significant improvements derived from taking HK-PS23 in job satisfaction or anxiety states, which was not received from the placebo group. This work demonstrates how HK-PS23 can have an anxiolytic effect, also shown by the reduction in serum cortisol levels, especially in groups of subjects accustomed to work under pressure and with high stress levels.

In 2023, Morales-Torres et al. [25] performed a double-blind, randomized, placebo-controlled study in Chile on many healthy adult volunteers. The study involved the treatment for 4 weeks of POS comprising 3 billion CFU of *Lactobacillus helveticus* R0052 and *Bifidobacterium longum* R0175 on 135 patients (80% women), aged between 20 and 66 years, divided into a treatment group and a placebo group. Stool samples were taken, and questionnaires were administered to assess the psychological state, effects on well-being, quality of life, emotional regulation, anxiety and interoceptive awareness. It was then investigated whether lifestyle behaviors modulated the efficacy of probiotics, but the results showed no significant effects of probiotic intake on the entire test population. A significant correlation was definitely demonstrated between the adoption of a healthy lifestyle and psychological well-being assessed on all scales. The application of a three-way interaction model between healthy behavior, treatment time and treatment group, corrected with the False Discovery Rate (FDR), reveals a significant interaction between healthy behavior and probiotic protective effects. Although this very recent study found no significant direct effect of psychobiotic use on psychological well-being, it highlights how adopting healthy habits could enhance the beneficial effect of these bacterial strains.

### 3.3. Mild Cognitive Impairment

Another application of psychobiotic is in the cognitive dysfunction treatment. Cognitive health can be affected by different factors, such as aging processes, mood disorders, psychological trauma, drug abuse, smoking, alcohol consumption, lifestyle (diet, physical exercise). In particular, researchers are studying the role of the diet and nutrition factors on cognitive decline. Hence, they are opening the door to new ways for treating this condition, wherein a low-fat diet and the supplement of vitamins, iron and polyphenols seem to be protective. Probiotics have another important role in the management of the gut microbiota composition with the eubiotic gut having beneficial effects on the brain through the gut-brain axis [26,27].

Aljumaah et al. [28] studied the use of psychobiotics on mild cognitive impairment, setting up a double-blind, randomized controlled trial on 169 middle-aged patients, which were divided into two groups: the placebo arm, 83 patients, and the probiotic arm, 86 patients. The tested probiotic was the *Lacticaseibacillus rhamnosus* GG (10 billion CFU), which is already known for its beneficial effects in the gut, inflammation and cognitive function, meanwhile the placebo consists of microcrystalline cellulose, an inert substance. At baseline and after three months of treatment, all the patients underwent cognitive and psychological evaluations. Researchers used the National Institutes of Health (NIH) Toolbox to test the neurological and behavioral functions, and they tested the memory, attention, and learning ability. Stool samples were also collected at baseline and after the supplementation for metagenomic analysis of the gut microbiota. After the psychobiotic supplementation, there was an improvement in the neuropsychological performance and the gut microbiota composition, for example, in mild cognitive impairment (MCI) subjects an abundance of the genus *Prevotella* was observed before the study, which decreases after the treatment.

### 3.4. Mood Disorders

Mood disorders are common psychiatric disorders, which lead to an increase in morbidity (e.g., anxiety, behavioral alterations, aggressivity, etc.) and mortality. The causes of onset can be different, and the clinical presentation, as well. Mood disorders have a refractory course, and this leads to some repercussion on the rehabilitative iter and long-term effects. The structural and functional alteration of neural circuits of emotions has been observed in this type of pathology, leading to dysfunction in their expression [29]. In the last few years, a growing interest was observed in the correlation between mood disorders and dysbiotic gut microbiota, suggesting that the gastrointestinal alteration can be fundamental in the development of neuropsychiatric disorders [30]. For this reason, it should be useful to support the classical treatment, consisting of psychotherapy and pharmacological treatment (antidepressant and mood-stabilizing medicine), with psychobiotic supplementation.

In a 2022 randomized, double-blind, placebo-controlled trial, Mutoh et al. [31] randomized a total of 58 healthy young adults, among third year nursing students under temporary stress prior to practical preparation, to receive either heat-killed *Lactobacillus helveticus* MCC1848 (5 × 10^9^) powder (29 subjects) or placebo (29 subjects) daily for 4 weeks. The authors used the short version of the Profile of Mood States 2 (POMS 2) score to determine the primary outcome measure of the study, as the type of mood improved by psychobiotics varies between strains. Secondary outcomes included other mood states, quality of life, sleep and fatigue as assessed by scores on the Athens Insomnia Scale (AIS), the STAI, the acute form of the SF-36v2, the Chalder Fatigue Scale (CFS), and VAS. At the end of the study, the results showed that consuming heat-killed *L. helveticus* MCC1848 for four weeks significantly improved “friendliness” scores and showed a tendency for improvement in “vigour-activity” scores based on POMS 2 measures, while the five negative scales did not seem to improve. The results suggest that heat-killed *L. helveticus* MCC1848 may selectively affect a positive mood. The study also looked at sleep and fatigue. However, using an intention-to-treat (ITT) approach, no changes were found in these measures with heat-killed *L. helveticus* MCC1848.

### 3.5. Eating Disorders

Eating disorders are disabling, damaging, deadly, and expensive mental disorders. They impair physical health and psychosocial functioning. A key role in the origin and maintenance of eating disorders is played by disturbances towards weight, body shape, and eating attitudes [32].

Obesity (the excess of body fat) is the result of a continuous and excessive calorie intake compared to a habitual consumption. A number of genetic, physiological, behavioral, and environmental factors contribute to the development of obesity. For this reason, obesity is not considered a mental disorder, but is strongly associated with (e.g., binge eating, schizophrenia, depressive disorder) [16].

Carlos et al. [14] tested the efficacy of psychobiotics on binge eating and food addiction disturbances on patients who underwent Roux-en-Y gastric bypass surgery. They set a double-blind, randomized controlled trial, which was conducted from April 2018 to December 2019. One hundred and one patients were initially selected, but only 44 were enrolled in the study and divided into the placebo arm and the psychobiotic one; both groups had to take for ninety days (starting from the seventh postoperative day) two tablets/day, but the placebo arm had an inert substance, instead of the other one which supplements the diet with a psychobiotic comprising 5 billion *Lactobacillus acidophilus* NCFM strain and 5 billion *Bifidobacterium lactis* Bi-07. Both groups received the same dietary orientations, had clinical follow-up assessments at T0 (10 days before the surgery), T1 (3 months later), T2 (one year later), and self-administered questionnaires, such as the Binge Eating Scale (BES) and Yale Food Addiction Scale (YFAS). By all these assessments, it was demonstrated at the end of the study that the early psychobiotic supplementation seems to decrease the symptoms correlated to binge eating and food addiction one year after the bariatric surgery.

De Lorenzo et al. [33] investigated the effects of psychobiotics on body composition and the related psychological profile in normal weight obese (NOW: BMI < 25 kg/m^2^ and a high total body fat percentage, TBFat > 30%) and obese patients. For this purpose, the team set up a double-blind, randomized controlled clinical trial, from October 2015 to July 2016. First, they selected 60 female patients, but they enrolled only 48 of them, which were subsequently divided into two groups. For the first 3 weeks, one group had to take a POS, made up of 1.5 × 10^10^ CFU of *Streptococcus thermophilus* SGSt01, *Bifidobacterium animalis* subsp. *lactis* SGB06, *Streptococcus thermophiles*, *Bifidobacterium bifidum* SGB02, *Lactobacillus delbrueckii* subsp. *bulgaricus* DSM 20081, *Lactococcus lactis* subsp. *lactis* SGLc01, *Lactobacillus acidophilus* SGL11, *Lactobacillus plantarum* SGL07, and *Lactobacillus reuteri* SGL01, meanwhile the other group had to take a placebo, made up of an inert material, totally indistinguishable from the POS. After the first stage, there was a wash-out period of 3 weeks, then, in conclusion, in the last 3 weeks each group reverses the treatment. Each arm had a clinical and psychological assessment at the beginning and the end of the study, where the participants underwent some questionnaires and tests, such as the SCL-90R, BUT, image perception (IC), and eating disorder inventory (EDI-2). The intake of psychobiotics by that suspension seems to have great effects on the psychological state and eating disorder, and overall clinical state.

### 3.6. Sleep Quality and Insomnia

Sleep disorders are really common in older adults, but they can be present also during adolescence. These disorders are commonly associated with an unhealthy status; indeed, they have an impact on overall health, mood, behavior, and social or professional performances [34,35]. Currently, there is a growing prevalence of sleep disorders and insomnia worldwide, and maybe they are linked to a dysbiotic gut microbiota [36]. The metabolites produced by the components of the gut microbiota, have an impact on brain function, behavior and generally human health; for this reason, dysbiosis is associated with an altered production of metabolites, generating consequently dysfunctions in all the parts cited above [37]. The growing prevalence of these disturbances makes the development of new treatments indispensable [38]. Melatonin and benzodiazepines are normally used to treat insomnia, but there is a growing interest in the psychobiotic supplement, especially in *Bifidobacterium,* with all the genera first grouped in the *Lactobacillus* genus.

Lan et al. [39] explored the use of psychobiotic, in particular, 5 × 10^9^ CFU *Bifidobacterium breve* CCFM1025, in the improvement of sleep quality. In the clinical trial, 66 patients were selected, but only 60 were enrolled and divided into three arms: 20 in the psychobiotic arm, 20 in the placebo one, and the last 20 constituted the healthy controls. All of them, except for the healthy controls, received a daily sachet of psychobiotic/placebo for four weeks; the results of this intervention were collected through the Pittsburgh Sleep Quality Index (PSQI) and AIS, which were administered at baseline and at the end of the study. Other tests were the measurement of salivary and plasma cortisol. From this study, the results revealed that the psychobiotic intake improves the sleep quality, justified by the reduction in PSQI and AIS, meanwhile the variation in cortisol concentration was not statistically significant. For these reasons, *Bifidobacterium breve* is an optimal candidate for enhancing sleep quality.

Zhu et al. [40] studied the role of a fixed dosage of 1.5 × 10^10^ CFU of *Lactiplantibacillus plantarum* JYLP-326 on anxiety, depression and insomnia symptoms, by testing anxious college students, who were preparing for an important entry exam. In the randomized controlled trial, 230 students were selected, but only 90 were enrolled and divided into three groups: 30 in the probiotic group, 30 in the placebo one, and the last 30 constituted the healthy control, because they do not suffer from an anxiety disorder. The two tested groups had to supplement their diet twice per day for three weeks with a probiotic/placebo product. The participants also had to answer some questionnaires, such as AIS, HDRS and HAM-A at baseline and at the end of the trial to compare the differences between the treatments. The researchers also asked for stool samples at the beginning and at the end of the trial, with the aim to study the gut microbiota composition and its changes and the faecal metabolism. At the end of the trial, the results suggested that supplementation with *Lactiplantibacillus plantarum* reduced symptoms of anxiety, depression and insomnia and contributed to a state of eubiosis of the gut microbiota. Therefore, this microorganism can be used as an effective psychobiotic for anxiety disturbances and the regulation of gut microbiota and faecal metabolites.

### 3.7. Stress

Stress is a common experience known for disrupting an organism’s balance, triggering adaptive physiological responses, particularly impacting the gastrointestinal (GI) tract and immune system [41,42]. Recent research underscores stress influence on GI processes such as gastric secretion, gut motility, and mucosal integrity, alongside its capacity to alter gut microbiota dynamics, impacting bacterial proliferation and virulence. The brain-gut-microbiota axis emerges as pivotal in mediating these effects, with stress-induced disturbances impacting the enteric nervous system (ENS), HPA, and neural connections forming the brain-gut axis (BGA) [43,44]. The corticotropin releasing factor (CRF) plays a critical role, influencing gut inflammation, permeability, and sensitivity [45].

Clinically, stress-related dysregulation of the BGA correlates with upper GI disorders including gastroesophageal reflux disease (GERD), peptic ulcer disease (PUD), inflammatory bowel diseases (IBDs) [46,47], and irritable bowel syndrome (IBS) [48,49]. Treatment strategies for these stress-related disorders include psychological therapies and lifestyle modifications [50].

Kelly et al. [51] employed a randomized, placebo-controlled, cross-over design to investigate the psychobiological effects of *Lacticaseibacillus rhamnosus* JB-1 (1 × 10^9^ CFU) on cognitive performance, immune response, and brain activity under acute stress. A total of 29 healthy male volunteers, aged 20 to 33 were included, ensuring sample homogeneity through strict exclusion criteria. Assessments were conducted at baseline, 4 weeks, and 8 weeks, with participants undergoing neurocognitive and acute stress visits utilizing the socially evaluated cold pressor test (SECPT). Each participant received either placebo or *L. rhamnosus* capsules for four weeks before switching treatments. Cognitive assessments utilized the Cambridge Neuropsychological Test Automated Battery (CANTAB), while EEG recordings evaluated brain activity patterns. The analysis involved cortisol and cytokine sampling, along with the Toll-like receptor 4 (TLR-4) cytokine release assessment. Statistical analyses revealed no significant overall effects on subjective stress measures or immune response. However, improvements in cognitive tasks were observed following both placebo and probiotic treatments. The EEG analysis indicated a significant difference in F3 zero crossings between placebo and probiotic phases. Overall, the study offers insights into the potential psychobiological benefits of *L. rhamnosus* JB-1, emphasizing improvements in cognitive performance and brain activity patterns under acute stress.

Nishida et al. [52] investigated the impact of a tablet containing heat-inactivated *Lactobacillus gasseri* CP2305 (1 × 10^10^ bacterial cells per two tablets) as a stress-relieving para-psychobiotic in 60 Japanese medical students facing chronic stress during their preparation for national medical exams. The study, conducted over nine months, utilized a rigorous double-blind, placebo-controlled trial design with 60 participants that were randomly assigned to either the CP2305 or placebo group and instructed to take two tablets per day for 24 weeks. The tablets, which were allergen-free, differed only in the presence of CP2305. Various assessments, including questionnaires on mental and physical health, were conducted throughout the study period. Weekly monitoring of stress-related symptoms, measurement of salivary cortisol and chromogranin A levels, sleep EEG analysis, and analysis of stool properties and gut microbiome changes were also performed. The results demonstrated that the CP2305 intake significantly reduced trait anxiety scores and improved sleep quality compared to the placebo. Moreover, CP2305 effectively alleviated stress-related symptoms such as irritability and abdominal discomfort. The sleep EEG analysis revealed enhancements in sleep latency and wake time after sleep onset with the CP2305 intake. Additionally, CP2305 significantly lowered salivary chromogranin A levels, indicating a reduction in stress response. While stool properties showed minimal changes, CP2305 mitigated stress-induced reductions in *Bifidobacterium* abundance and prevented the elevation of *Streptococcus* in the gut microbiome. Furthermore, the CP2305 intake increased faecal concentrations of n-valeric acid, suggesting a positive impact on gut health. Overall, these findings suggest that CP2305 may offer beneficial effects on mental and physical well-being, potentially through its modulation of the gut microbiota and stress response pathways.

Allen et al. [53] aimed to determine if the effects seen in earlier animal studies with *Bifidobacterium longum* 1714 (1 × 10^9^ CFU) could be replicated in healthy human volunteers. They conducted a placebo-controlled trial involving 22 male participants, examining how the 1714 strain impacted stress, cognitive abilities, and brain activity. Participants were screened for mental health conditions and demographic details were collected. Using a repeated measures design, participants received either placebo or the 1714 strain for four weeks each, with assessments before, during, and after each phase. Daily online surveys gauged stress levels, while cognitive tests assessed memory, attention, social understanding, and emotional responses. The SECPT induced acute stress, with cortisol levels measured throughout. The results showed lower cortisol levels and reduced anxiety post-stress with 1714 supplementation. Daily stress was also lower during the 1714 phase, and overall stress levels were significantly reduced. Cognitive performance, especially in paired associate learning, improved after 1714 supplementation. EEG measurements indicated changes in brain activity, suggesting enhanced attention and arousal following 1714 supplementation. In summary, the study implies that *Bifidobacterium longum* 1714 could alleviate stress, enhance cognitive abilities, and influence brain function in healthy individuals.

### 3.8. Autism Spectrum Disorders

ASDs are heritable neurodevelopmental conditions characterized by social communication deficits, repetitive behaviors, and restricted interests [54], classified in the DSM-III as a pervasive developmental disorder [55]. The ASD diagnosis is based on observing behavior according to the DSM-5 criteria, as there are no reliable biological markers for this condition [56]. ASD often occurs alongside other conditions like attention deficit hyperactivity disorder (ADHD), anxiety, and genetic disorders [57]. The prevalence of ASD is increasing, but disparities exist in early diagnosis rates across ethnical groups [58]. Therapeutic approaches for ASD can be different [59,60,61] and are individualized, with the aim of improving the patient’s quality of life.

Liu et al. [62] aimed to explore how *Lactiplantibacillus plantarum* PS128, provided in a capsule containing 3 × 10^10^ CFU, might positively affect ASD symptoms. The study, employing a double-blind, randomized, parallel, placebo-controlled design, involved 80 boys aged 7 to 15 diagnosed with ASD. Various assessment tools, including the Autism Behavior Checklist-Taiwan version (ABC-T), Social Responsiveness Scale (SRS), Child Behavior Checklist (CBCL), Clinical Global Impression (CGI), and Swanson, Nolan, and Pelham-IV (SNAP-IV), were used to evaluate subjects at baseline and week 4. The results revealed no significant differences in CGI-I scores between the PS128 and placebo groups. While total ABC-T or SRS scores did not show significant differences, there was a trend indicating that PS128 might reduce body and object use scores on ABC-T and total SRS scores. Moreover, PS128 showed nominal reductions in CBCL anxiety and rule-breaking behaviors, as well as SNAP-IV total scores, hyperactivity/impulsivity, and opposition/defiance. Upon further analysis by age groups (7–12 and 13–15 years), improvements were observed in social awareness, opposition/defiance, and SNAP-IV scores among younger participants receiving PS128. These findings suggest that PS128 holds promise in alleviating specific ASD symptoms, particularly in younger individuals.

Table 1 provides a comparison of all the studies reviewed in this paper, based on the bacterial strains that were used and the results obtained.

**Table 1:** The studies analyzed are summarized schematically, dividing them by pathology and indicating the bacterial species used and the results obtained.

Pathology	Paper	Bacterial Strain Administered	Results
depression	Tian et al. (2021) [19]	*Bifidobacterium breve* CCFM1025	The psychotropic potential of *B. breve* CCFM1025 was evaluated in MDD patients. As an adjunctive supplement, the intake of CCFM1025 significantly, and to a more considerable extent, reduced the patients’ psychiatric and gastrointestinal symptoms compared with the placebo. The effect may be correlated with the change in gut microbiome and tryptophan metabolism.
	Karakula-Juchnowicz et al. (2019) [18]	*Lactobacillus helveticus* R0052 and *Bifidobacterium longum* R0175	The combination of gluten-free diet and probiotic supplementation can stop the immune-inflammatory cascade in the MDD course and enhance both psychiatric and gut barrier-associated features.
	Zhu et al. (2023) [40]	*Lactiplantibacillus plantarum*	The administration of this psychobiotic in anxious college students preparing for an important exam led to a reduction in symptoms related to anxiety, depression and insomnia, as they have declared in the self-administered questionnaires. An improvement in the composition of gut microbiota by collecting stool samples was also observed.
	Rudzki et al. (2018) [17]	*Lactiplantibacillus plantarum* 299v	The implementation of LP 299v to SSRI therapy enhanced cognitive performance and reduced KYN concentrations in MDD patients.
anxiety	Morales-Torres et al. (2023) [25]	*L. helveticus*, *B. longum*	No significant effect of probiotics on psychological well-being, but the adoption of healthy habits could enhance the beneficial effect of probiotics.
	Tran et al. (2019) [22]	OTC Probiotics	Post hoc analyses revealed that the CFU level was more effective than the species number in achieving significant improvements. A ceiling effect was found in the study; participants with high discomfort reported more improvements than those with discomfort in the normal range.
	Wu et al. (2022) [24]	HK-PS23 (Heat-Killed *Lactobacillus paracasei* PS23)	HK-PS23 showed potential benefits in reducing cortisol levels compared with the placebo and could improve perceived anxiety states in nurses with particularly high stress levels.
	Colica et al. (2017) [21]	*S. thermophilus*, *L. bulgaricus*, *L. lactis* subsp. *lactis*, *L. acidophilus*, *S. termofili*, *L. plantarum*, *B. lactis*, *L. reuteri*	The use of probiotics (POS) generated a reduction in anxiety-related test scores, to a greater extent for highly anxious subjects.
	Gualtieri et al. (2020) [23]	*S. thermophilus*, *B. animalis* subsp. *lactis*, *B. bifidum*, *S. thermophiles*, *L. bulgaricus*, *L. lactis* subsp. *lactis*, *L. acidophilus*, *L. plantarum*, *L. reuteri*	Consumption of probiotics mitigates anxiety symptoms, especially in healthy adults with the A allele of rs16944 as a risk factor (IL-1 β polymorphism), who had a significant reduction in HAM-A score and the frequency rate of anxious carriers was reduced. Clinical evidence is lacking in defining which probiotic strains clearly have psychobiotic properties.
mild cognitive impairment	Aljumaah et al. (2022) [28]	*Lacticaseibacillus rhamnosus*	After the psychobiotic admnistration, the MCI patients reported an improvement in the neuropsychological performances and the gut microbiota composition, in particular, a reduction in the genus *Prevotella* was observed.
mood disorders	Mutoh et al. (2022) [31]	*Lactobacillus helveticus* MCC1848	Consuming heat-killed *L. helveticus* MCC1848 for four weeks significantly improved “friendliness” scores and showed a tendency for improvement in “vigour-activity” scores based on POMS 2 measures, while the five negative scales did not seem to improve. Heat-killed *L. helveticus* MCC1848 may selectively affect a positive mood. The study also looked at sleep and fatigue. However, using an intention-to-treat (ITT) approach, no changes were found in these measures with heat-killed *L. helveticus* MCC1848.
	Karakula- Juchnowicz et al. (2019) [18]	*Lactobacillus helveticus* R0052 and *Bifidobacterium longum* R0175	The combination of gluten-free diet and probiotic supplementation can stop the immune-inflammatory cascade in the MDD course and enhance both psychiatric and gut barrier-associated features.
	De Lorenzo et al. (2017) [33]	*Streptococcus thermophilus*, *Bifidobacterium animalis* subsp. *lactis*, *Streptococcus thermophiles*, *Bifidobacterium bifidum*, *Lactobacillus delbrueckii* subsp. *bulgaricus*, *Lactococcus lactis* subsp. *lactis*, *Lactobacillus acidophilus*, *Lactobacillus plantarum*, and *Lactobacillus reuteri*	The psychobiotics intake (in POS form) had a positive effect on NOW and obese female patients. They reported in the self-administered questionnaires an improvement in the psychological state and eating disorders, and a physical improvement in the clinical assessment was confirmed.
eating disorders	Carlos et al. (2022) [14]	*Lactobacillus acidophilus*, *Bifidobacterium lactis*	The patients who underwent baritric surgery reported in the self-administered questionnaires, after the psychobiotic supplementation, an improvement in the symptoms correlated to binge eating and food addiction.
	De Lorenzo et al. (2017) [33]	*Streptococcus thermophilus*, *Bifidobacterium animalis* subsp. *lactis*, *Streptococcus thermophiles*, *Bifidobacterium bifidum*, *Lactobacillus delbrueckii* subsp. *bulgaricus*, *Lactococcus lactis* subsp. *lactis*, *Lactobacillus acidophilus*, *Lactobacillus plantarum*, and *Lactobacillus reuteri*	The psychobiotics intake (in POS form) had a positive effect on NOW and obese female patients. They reported in the self-administered questionnaires an improvement in the psychological state and eating disorders, and a physical improvement in the clinical assessment was confirmed.
	Colica et al. (2017) [21]	*S. thermophilus*, *L. bulgaricus*, *L. lactis* subsp. *lactis*, *L. acidophilus*, *S. termofili*, *L. plantarum*, *B. lactis*, *L. reuteri*	The use of probiotics (POS) generated a reduction in anxiety-related test scores, to a greater extent for highly anxious subjects.
sleep quality and insomnia	Lan et al. (2023) [39]	*Bifidobacterium breve*	After the psychobiotics supplementation of four weeks, the patients of the experimental arm reported an improvement in sleep quality in the self-administered questionnaires, even if the levels of cortisol were not significantly decreased.
	Zhu et al. (2023) [40]	*Lactiplantibacillus plantarum*	The administration of this psychobiotic in anxious college students preparing for an important exam led to a reduction in symptoms related to anxiety, depression and insomnia, as they declared in the self-administered questionnaires. An improvement in the composition of gut microbiota was also observed by collecting stool samples.
stress	Kelly et al. (2016) [51]	*Lactobacillus rhamnosus* JB-1	The intake of *Lactobacillus rhamnosus* (JB-1) as a psychobiotic did not show significant effects on mood, anxiety, stress, or sleep quality compared to a placebo. It also had no impact on cognitive performance, such as memory, attention, or emotion recognition, and did not alter the inflammatory response or stress hormone levels.
	Nishida et al. (2019) [52]	*Lactobacillus gasseri* CP2305	The long-term intake of *Lactobacillus gasseri* CP2305 in tablet form improved stress-associated symptoms in young adults, particularly in reducing trait anxiety scores. While no effect on basal salivary cortisol levels was observed, the CP2305 tablet significantly reduced salivary chromogranin A (CgA) levels, a marker of stress.
	Allen et al. (2016) [53]	*Bifidobacterium longum* 1714	*Bifidobacterium longum* 1714 has demonstrated promising effects as a psychobiotic, reducing both physiological and psychological responses to stress in healthy humans. The intake of this strain resulted in lower cortisol levels during acute stress and decreased self-reported anxiety. Additionally, subtle improvements in visuospatial memory and enhanced prefrontal cortex activity, as indicated by EEG, suggest cognitive benefits.
	Wu et al. (2022) [24]	HK-PS23 (Heat-Killed *Lactobacillus paracasei* PS23)	HK-PS23 showed potential benefits in reducing cortisol levels compared with the placebo and could improve perceived anxiety states in nurses with particularly high stress levels.
autism spectrum disorders	Liu et al. (2019) [62]	*Lactobacillus plantarum* PS128	*Lactobacillus plantarum* PS128 showed significant benefits for boys with autism spectrum disorder (ASD), particularly in reducing opposition and defiance behaviors. Younger children (ages 7–12) experienced notable improvements in SNAP-IV scores compared to the placebo group. After 28 days of PS128 consumption, several behavioral aspects improved, suggesting its potential as a supportive intervention for ASD.

### 3.9. Activity Overview

**Figure 2:**
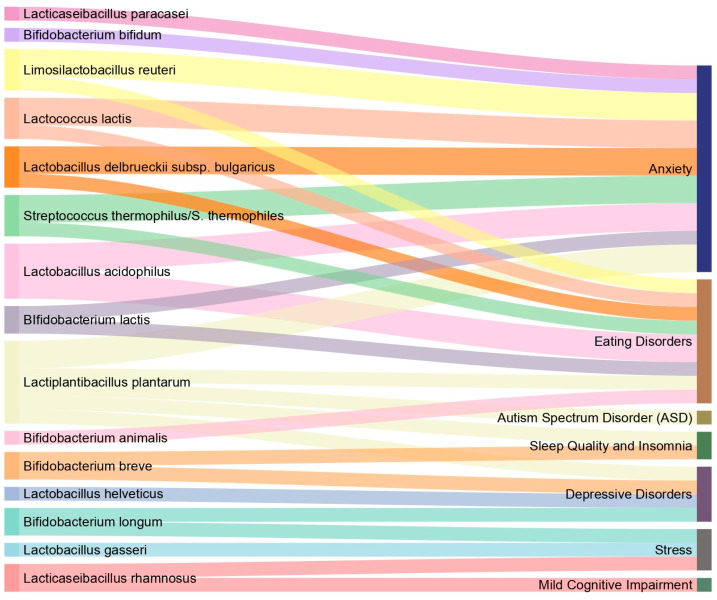
Sankey diagram linking the psychobiotics species and disorders for which they have been shown to have a positive effect in the studies reviewed. The thickness of the lines, corresponding to the bacterial species, is proportional to the number of studies in which they have been used for psychobiotic therapies that have shown positive results. For example, *Lactobacillus gasseri* species was used positively in only one study for the treatment of stress, while below, *Lactobacillus rhamnosus* provided positive effects in two studies: one for the treatment of stress and one for mild cognitive impairment.

Regarding the therapeutic or beneficial effects provided by the different bacterial species employed as psychobiotics, we investigated which strains were the most commonly used to treat the analyzed disorders. Considering only those studies in which a significant correlation was observed between the use of psychobiotics and disease improvement, in Figure 2 we connected the bacterial species to the respective disorders for which they had a positive effect. We can observe that *Lactiplantibacillus plantarum* contributes to improvements in several disorders such as anxiety, eating disorders, autism, insomnia, and depression. This finding certainly spotlights a bacterial species that, based on the studies reviewed, appears to contribute to the improvement in symptoms of a wide variety of diseases and could offer insights for further investigations.

### 3.10. Current Evidence

Psychobiotics are live microorganisms that confer mental health benefits through interactions with the gut microbiota and have emerged as a promising avenue for addressing various psychiatric and neurodevelopmental disorders. These microorganisms influence neural signaling and biochemical pathways crucial for mental health, such as those involving neurotransmitters and the HPA axis. While the concept holds promise, integrating psychobiotics into clinical practice needs rigorous exploration about their efficacy, mechanisms of action, and long-term safety.

Several studies have investigated the efficacy of psychobiotics across different conditions, including depression, anxiety, stress, sleep disorders, ASD, and alimentary disorders. Our investigation has provided valuable insights into psychobiotics therapeutic potential, mechanisms of action, limitations, and future research directions. With this systematic review of clinical trials or randomized controlled-trials (RCTs), our aim is to evaluate the efficacy of psychobiotics on the treatment of psychological disorders, major depression, anxiety, insomnia, stress, mild cognitive impairment, autism spectrum disorder, and eating disorders.

The research brought to our attention 18 manuscripts based on clinical trials, which tested the efficacy of psychobiotics in managing these psychiatric disorders.

It is important to underline that psychobiotics are not miraculous cures but their efficacy depends on other important factors such as a balanced diet; indeed, Morales-Torres et al. (2023) [25] have observed that a healthy eating habit improves the effect of psychobiotics on anxiety management. Studies on psychobiotics in depression have demonstrated improvements in depressive symptoms and related physiological parameters. Tian et al. (2021) [19] found that *Bifidobacterium breve* CCFM1025 led to significant reductions in depression symptoms and improvements in gastrointestinal health, indicating a potential role in modulating serotonin metabolism and gut microbiota diversity. However, variability in study designs, small sample sizes, and the lack of long-term follow-up limit the generalizability and comprehension of the effects of psychobiotics on depression.

Similarly, in anxiety disorders, psychobiotics like *L. helveticus* R0052 and *Bifidobacterium longum* R0175 have been promising in reducing anxiety symptoms, particularly when considering genetic predispositions and lifestyle factors (Gualtieri et al., 2020) [23]. However, the exact mechanisms through which these probiotics exert their effects on anxiety pathways remain unclear, necessitating further mechanistic studies and larger-scale trials.

In the context of ASD, Liu et al. (2019) [62] have explored the potential of probiotics like *L. plantarum* PS128 to improve specific behavioral aspects, such as oppositional behaviors in young children (aged 7–12). While these findings are encouraging, the limitations of small sample sizes and short intervention periods underscore the need for more extensive research to validate these outcomes across broader ASD populations and to elucidate underlying mechanisms.

Moreover, psychobiotics have shown positive results in managing alimentary disorders, such as obesity and eating disorders, by influencing body composition, eating behaviors, and psychological profiles (Carlos et al. (2022) [14], De Lorenzo et al. (2017) [33], Colica et al. (2017) [21]). Studies have highlighted significant improvements with post-surgery psychobiotic supplementation, suggesting potential roles in enhancing long-term outcomes and sustaining therapeutic benefits (Carlos et al., 2022) [14]. Nonetheless, study duration, sample size, and the need for a personalized treatment emphasize the complexity of translating these findings from animal models to clinical settings. According to the last point, Kelly et al. (2016) [51] in their trial found discrepancies between preclinical and human trials; indeed, the statistical analyses revealed no significant overall effects on subjective stress measures.

Probiotics are well-known support in a number of both infectious and chronic diseases, but only in recent years they have been taken into consideration to help individuals with psychiatric disorders. Our study highlights the main points of strength of the published clinical research in humans, and the important potential of psychobiotics in the future therapy protocols. However, we hope that the next studies can explore a more extensive and homogeneous population, and elucidate their molecular mechanisms of action, allowing for further understanding the gut-brain axis and CNS physiology.

## 4. Conclusions

Despite the above-discussed promising results, several critical challenges and limitations persist in psychobiotic research. Standardizing protocols across studies, validating efficacy through rigorous clinical trials with diverse populations, and understanding the long-term safety profiles are essential steps in advancing psychobiotics as mainstream therapeutic options. Additionally, comprehending the intricate interactions between psychobiotics, the gut microbiota, and the brain’s neural pathways are crucial for developing targeted interventions that optimize efficacy and ensure patient’s safety.

In conclusion, while psychobiotics represent a valid frontier in mental health and neurodevelopmental disorder treatments, further robust research is needed to consolidate their therapeutic effectiveness, to elucidate underlying mechanisms, and to address current study limitations. For these reasons, psychobiotics could potentially offer personalized and effective interventions to support existing treatments for a range of psychiatric and neurodevelopmental conditions. Finally, we must highlight these final considerations:

- Depression and gut microbiota-brain axis dysbiosis may be linked through the interaction of neuroimmune, neuroendocrine, and metabolic pathways mediated by the gut microbiota.
- Patients with psychiatric illnesses or congenital disorders may have different gut microbiota profiles compared to healthy controls, while patients with intestinal inflammation experience mood and cognitive impairment.
- Psychobiotics and prebiotics aid to improve these pathological conditions because they can reduce inflammation and rebalance the gut microbiota through the so-called pathways previously mentioned.
- Prebiotic-probiotic combination therapy in combination with antidepressants or other drugs for the treatment of diseases or disorders in this medical field has attracted attention as a potential therapeutic approach for mental disorders.

## Data Availability

Data are contained within the article.

## Acronyms

3HAA	3-hydroxy anthranilic acid
3HKYN	3-hydroxykynurenine
5-HT	serotonin
AA	anthranilic acid
ABA	Applied Behavior Analysis
ABC-T	Autism Behavior Checklist-Taiwan version
ACQ-R	anxiety control questionnaire-revised
ADOS	Autism Diagnostic Observation Schedule
ADHD	attention deficit hyperactivity disorder
AIS	Athens Insomnia Scale
APT	Attention and Perceptivity test
ASD	Autism Spectrum Disorder
BAI	Beck anxiety inventory
BBB	blood brain barrier
BDI	Beck’s Depression Inventory
BDNF	brain-derived neurotrophic factor
BES	Binge Eating Scale
BGA	brain-gut axis
BIA	bioelectrical impedance analysis
BMI	body mass index
BPRS	Brief Psychiatric Rating Scale
BUT	Body Uneasiness Test
CFU	colony forming unit
CANTAB	Cambridge Neuropsychological Test Automated Battery
CBCL	Child Behavior Checklist
CFS	Chalder Fatigue Scale
CGI	Clinical Global Impression
CNS	central nervous system
CRF	Corticotrophin releasing factor
CTG	combination treatment group
CVLT	California Verbal Learning Test
DTG	diet treatment group
DXA	dual X-ray absorptiometry
EDI-2	eating disorder inventory
EEG	Electroencephalography
EFSA	European Food Safety Authority
ENS	enteric nervous system
EPM	Elevated Plus Maze
ESDM	Early Start Denver Model
FDA	Food and Drug Administration
FDR	False Discovery Rate
GABA	gamma-amino butyric acid
GERD	gastroesophageal reflux disease
GF	germ-free
GI	gastrointestinal
GLP-1	glucagon-like peptide 1
GSRS	gastrointestinal symptom rating scale
HAM-A	Hamilton Anxiety Assessment Scale
HAM-D17	Hamilton Depression Rating Scale
HDRS-24	Hamilton Depression Rating Scale-24 items
HK	heat-killed
HPA	hypothalamic-pituitary-adrenal
IBD	inflammatory bowel diseases
IBS	irritable bowel syndrome
IC	image perception
IL-6	interleukin-6
IL-1β	interleukin 1- Beta
ISI	insomnia severity index
ITT	intention-to-treat
JSS	Job Stress Scale
KYN	Kynurenine
KYNA	Kynurenic acid
LPS	lipopolysaccharides
MADRS	Montgomery-Asberg Depression Rating Scale
MCI	Mild cognitive impairment
M-CHAT	Modified Checklist for Autism in Toddlers
MDD	Major Depressive Disorder
NIH	National institutes of health
NMR	negative mood regulation
NREM	Non-Rapid Eye Movement
NSAID	non-steroidal anti-inflammatory drugs
PANAS	positive and negative affect schedule
PCG	placebo control group
PHQ-9	Patient Health questionnaire
POMS 2	Profile of Mood States 2
POS	psychobiotic oral suspension
POSG	psychobiotic oral suspension group
PSS-10	Perceived Stress Scale
PSQI	Pittsburgh Sleep Quality Index
PSWQ	Penn state worry questionnaire
PUD	peptic ulcer disease
QLESQ-SF	Quality of life enjoyment and satisfaction questionnaire short form
RCT	randomized controlled-trials
RFFT	Riff Figural Fluency test
SCFA	short-chain fatty acids
SCL-90	Symptoms checklist
SECPT	socially evaluated cold pressor test
SF-36	Short Form Health Survey 36
SNAP-IV	Swanson, Nolan, and Pelham-IV
SNP	single nucleotide polymorphism
SRS	Social Responsiveness Scale
SSRI	serotonin reuptake inhibitors
STAI	State and Trait Anxiety Index
TBFat%	total body fat percentage
TMT	Trail Making test
TNF-α	tumor necrosis factor-alpha
TRP	tryptophan
VAS	visual analogue scale for gastrointestinal symptoms
YFAS	Yale Food Addiction Scale
